# Antireflective silicon nanostructures with hydrophobicity by metal-assisted chemical etching for solar cell applications

**DOI:** 10.1186/1556-276X-8-159

**Published:** 2013-04-08

**Authors:** ChanIl Yeo, Joon Beom Kim, Young Min Song, Yong Tak Lee

**Affiliations:** 1 School of Information and Mechatronics, Gwangju Institute of Science and Technology, 1 Oryong-dong, Buk-gu, Gwangju 500-712, South Korea; 2 Department of Nanobio Electronics and Materials, Gwangju Institute of Science and Technology, 1 Oryong-dong, Buk-gu, Gwangju 500-712, South Korea

**Keywords:** Silicon nanostructures, Metal-assisted chemical etching, Antireflection, Self-cleaning, Solar cell

## Abstract

We present broadband antireflective silicon (Si) nanostructures with hydrophobicity using a spin-coated Ag ink and by subsequent metal-assisted chemical etching (MaCE). Improved understanding of MaCE, by conducting parametric studies on optical properties, reveals a design guideline to achieve considerably low solar-weighted reflectance (SWR) in the desired wavelength ranges. The resulting Si nanostructures show extremely low SWR (1.96%) and angle-dependent SWR (<4.0% in the range of 0° to 60°) compared to that of bulk Si (SWR, 35.91%; angle-dependent SWR, 37.11%) in the wavelength range of 300 to 1,100 nm. Relatively large contact angle (approximately 102°) provides a self-cleaning capability on the solar cell surface.

## Background

Over the past decades, a great deal of efforts has been carried out to improve the conversion efficiency of crystalline silicon (c-Si) solar cells, which occupy most of the solar cell market [1,2]. To achieve a high-efficiency c-Si solar cell, antireflective layers/structures are inevitably necessary for enhancing the transmission of the sunlight into the solar cells by suppressing surface reflection, which is caused by the refractive index difference at the air/c-Si interface. Recently, subwavelength-scale nanostructures have attracted considerable attention as a promising antireflective structure to minimize unwanted reflection losses, due to their long-term stability, and broadband and omnidirectional antireflection properties [3-10]. To produce subwavelength-scale Si nanostructures, a dry etching method using nanoscale mask patterns has been commonly employed [7-10]. However, this method is complex, expensive, and inadequate for mass production and may cause damage to the crystal structure and surface morphology due to high-energy ions [11]. In recent years, metal-assisted chemical etching (MaCE), based on the strong catalytic activity of metal in an aqueous solution composed of HF and an oxidant, has attracted great interest as a method for fabricating Si nanostructures for electronic and optoelectronic devices [2,6,12-18]. This is a simple, fast, cost-effective, and high-throughput method for fabricating various Si nanostructures without any sophisticated equipment or ion-induced surface damages. The antireflection properties of nanostructures are strongly correlated with their dimensions and etching profiles [4-8], which can be controlled by adjusting the pattern of the metal catalyst [6] and etching conditions, such as etching time, etchant concentration, and etching temperature for MaCE [6,12-16]. However, the antireflection characteristics of Si nanostructures, which take into account the etchant concentration and etching temperature of MaCE, have been less explored. Therefore, it is meaningful to investigate the optimum Si MaCE condition to achieve desirable antireflective Si nanostructures for practical solar cell applications. Another aspect of this parametric study is that we could confirm the self-cleaning effects of the fabricated structures as well as the optical properties [19].

In this paper, we investigated the influence of Si MaCE conditions including the concentration of HNO_3_ (i.e., oxidant), HF, deionized (DI) water, and etching temperature on the morphologies and optical properties of Si nanostructures for obtaining the most appropriate antireflective Si nanostructures with self-cleaning function for solar cell applications. Antireflection properties of the fabricated Si nanostructures were systematically investigated by hemispherical reflectance spectrum measurement and calculating solar-weighted reflectance (SWR). The surface wetting behavior of the Si nanostructures was also analyzed by the water contact angle measurement.

## Methods

**Figure 1:**
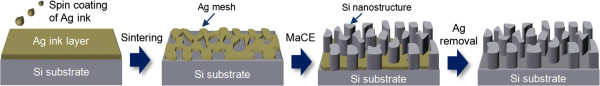
The process steps to fabricate Si nanostructures using spin-coated Ag ink and by subsequent MaCE.

Figure 1 shows a schematic illustration of the process procedures for fabricating Si nanostructures on a single-side-polished Si substrate (p-type (100), 1 to 30 Ω cm, approximately 25 × 25 mm^2^) by MaCE with spin-coated Ag mesh patterns [6]. Details of the spin-coated Ag ink and explanation of the experimental process can be found in the literature [6]. In this work, an aqueous solution containing HNO_3_ (70%), HF (50%), and DI water was utilized. The HNO_3_ was used as an oxidant to selectively oxidize the Si underneath the Ag mesh patterns by providing positive holes (h^+^) into Si instead of H_2_O_2_ and AgNO_3_, which have been widely explored for Si MaCE [12-18]. In order to produce Si nanostructures with reasonable height, the etching time was fixed as 450 s because nanostructures with extremely tall height can be bunched together and may be mechanically unstable [4,13]. To investigate the influence of the concentration of etch solution on the morphologies and optical properties of the fabricated Si nanostructures, the quantity of target etchant was adjusted while fixing the quantity of other etchants and the etching temperature (23°C). The effect of etching temperature on the morphologies and optical properties of the resulting Si nanostructures was investigated with a fixed quantity of HNO_3_, HF, and DI water. All variables for the Si MaCE process were carefully adjusted to obtain a suitable etching rate and morphology for solar cell applications [15]. After the Si MaCE process, the residual Ag was completely removed by immersing the samples in a wet etchant containing KI, I_2_, and DI water (KI/I_2_/DI = 1 g:1 g:40 ml) for 5 s at room temperature without any change in the shape of Si nanostructures; this was followed by rinsing with DI water and drying with N_2_ jet.

## Results and discussion

**Figure 2:**
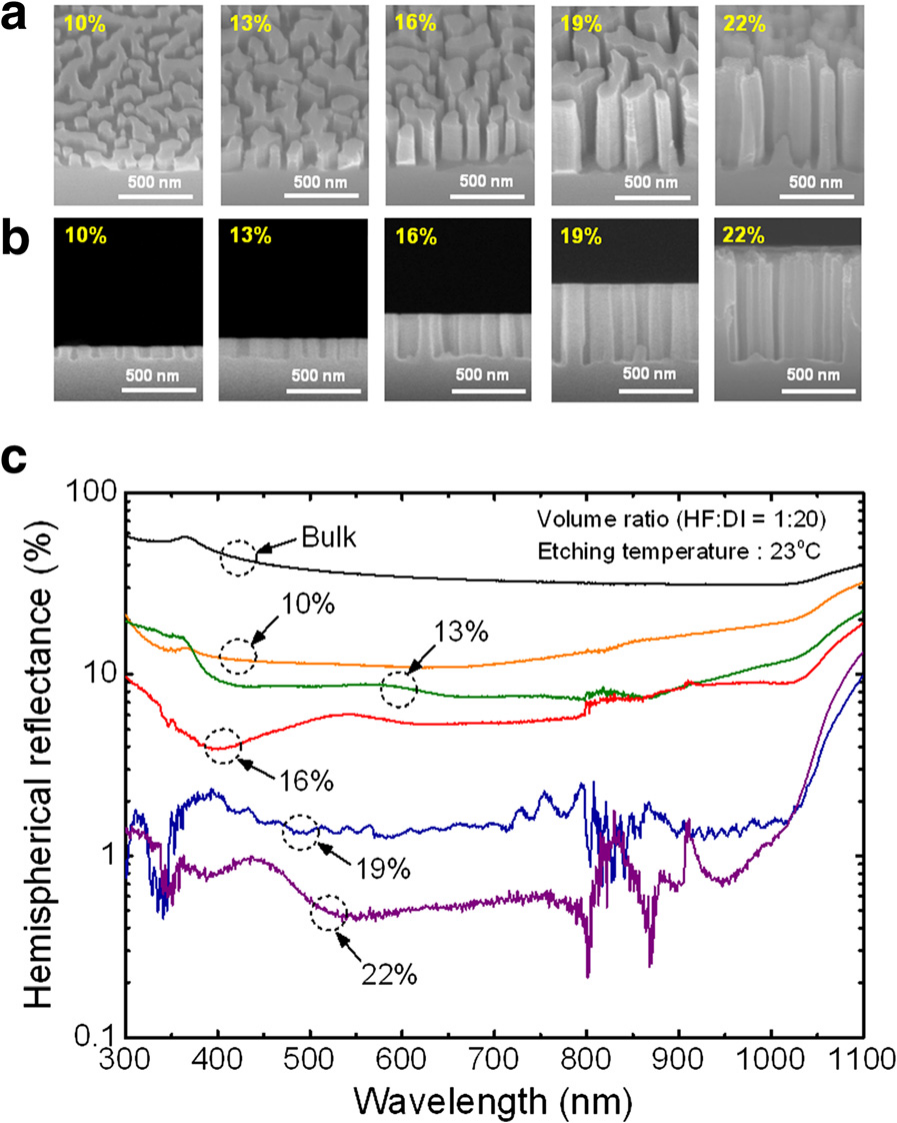
**SEM images of the Si nanostructures and measured hemispherical reflectance spectra.** (**a**) 45° tilted- and (**b**) cross-sectional-view SEM images of the Si nanostructures fabricated using different HNO_3_ concentrations from 10% to 22% in an aqueous solution. (**c**) Measured hemispherical reflectance spectra of the corresponding Si nanostructures as a function of wavelength.

Figure 2 shows the influence of HNO_3_ concentration on the morphologies and antireflection properties of the produced Si nanostructures. The HNO_3_ concentration was adjusted from 10% to 22% in an aqueous solution, which was composed of HF and DI water with a fixed volume ratio (1:20 *v*/*v*), by pouring in additional HNO_3_. The field-emission scanning electron microscope (FE-SEM, S-4700, Hitachi, Ltd., Tokyo, Japan) images clearly reveal that the average height of the Si nanostructures increases from 96 ± 14 to 695 ± 47 nm and the etching rate of Si nanostructures increases from 12.8 to 92.7 nm/min by increasing the HNO_3_ concentration. The increase of etching rate is due to the faster oxidation of Si as the HNO_3_ concentration increases because the amount of positive holes (h^+^) injected into Si, giving rise to the oxidation of Si, depends on the concentration of oxidant [12]. Figure 2c shows the measured hemispherical reflectance spectra of the corresponding Si nanostructures in the wavelength range of 300 to 1,100 nm, which cover the primary solar energy spectrum that is of interest in Si solar cells. The reflectance of the bulk Si is also shown as a reference. The hemispherical reflectance spectra were measured using a UV–VIS-NIR spectrophotometer (Cary 500, Varian, Inc., Palo Alto, CA, USA) equipped with an integrating sphere at the near-normal incident angle of 8°. The Si nanostructures remarkably reduced the reflection compared to that of the bulk Si (>30%) over the entire wavelength range of 300 to 1,100 nm. As the HNO_3_ concentration increases, the hemispherical reflectance gradually decreases due to the increased height of the Si nanostructures. It is well known that nanostructures with taller height exhibit better antireflection properties [3-7]. To investigate the effective reflection of the Si nanostructures on the solar cell performance under the solar radiation spectrum (i.e., the terrestrial air mass 1.5 global (AM 1.5G) [20]), we calculated the SWR, as given in the following equation [21]:

$$\mathrm{SWR}=\frac{\int \;R\left(\lambda \right)N_{\mathrm{photon}}\mathrm{d\lambda}}{\int \;N_{\mathrm{photon}}\mathrm{d\lambda}},$$

where *R*(*λ*) is the reflectance and *N*_photon_ is the photon number of AM 1.5G per unit area per unit wavelength. As the HNO_3_ concentration increased, the SWR of the Si nanostructures was decreased from 13.44% to 0.92%, which was a much lower value than the polished surface (35.91%), in the wavelength range of 300 to 1,100 nm. Although the Si nanostructures fabricated using an HNO_3_ concentration of 22% demonstrated the lowest SWR compared to other conditions, excessive HNO_3_ concentration can generate a rough morphology which can deteriorate the performance of solar cells because of considerable surface states (i.e., trap photo-generated carriers) and the challenge in forming ohmic contacts [10], as can be seen in Figure 2a,b. Hence, proper concentration of oxidant is required to produce desirable Si nanostructures, with a smooth and flat surface, by MaCE process for solar cell applications.

**Figure 3:**
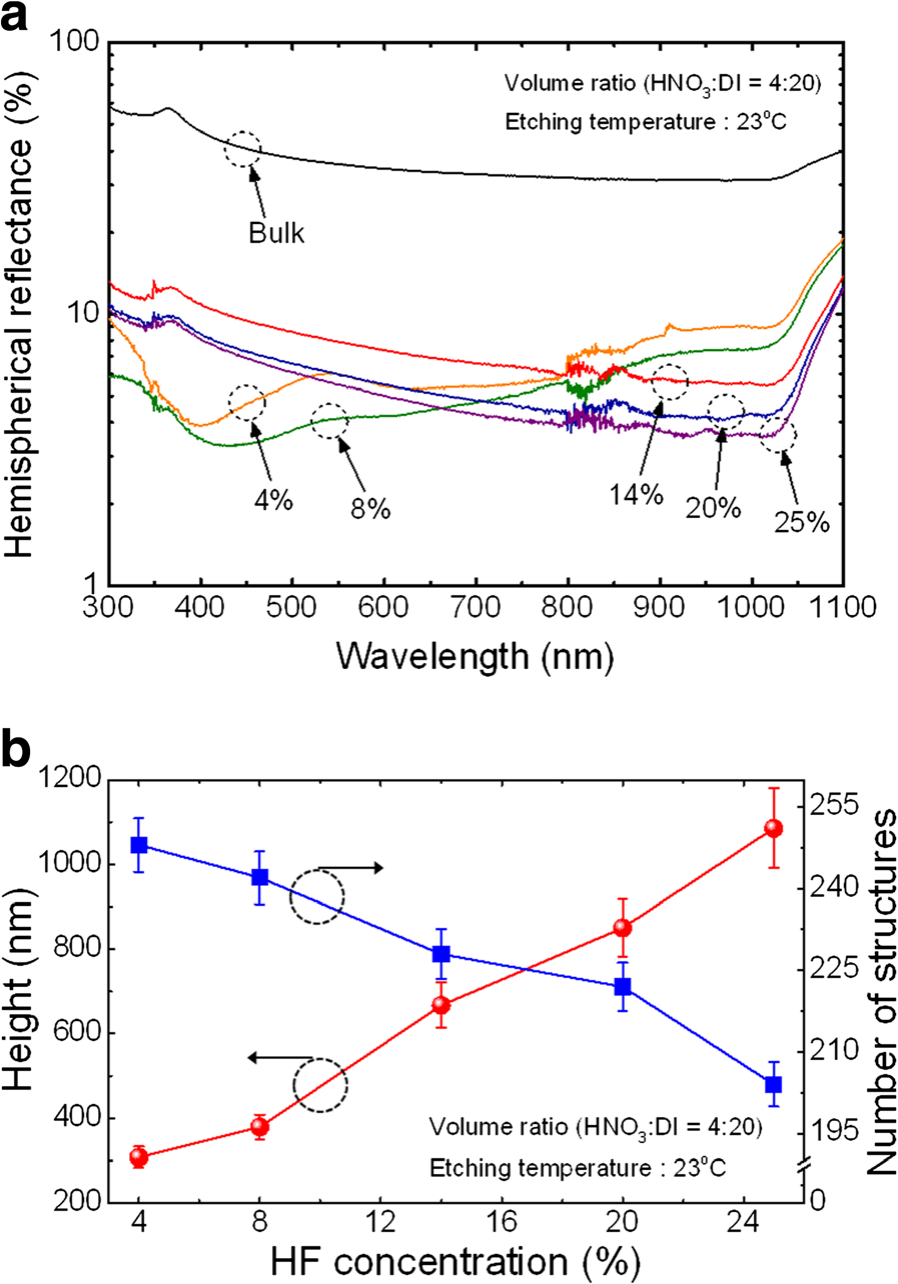
**Measured hemispherical reflectance spectra and estimated average height and number of structures.** (**a**) Measured hemispherical reflectance spectra of the Si nanostructures fabricated using different HF concentrations from 4% to 25% in an aqueous solution. (**b**) Estimated average height and number of structures within a unit area as a function of HF concentration.

Figure 3a shows the HF concentration-dependent hemispherical reflectance spectra of Si nanostructures in the wavelength range of 300 to 1,100 nm. The HF concentration was adjusted from 4% to 25% in an aqueous solution, which contained HNO_3_ and DI water with a fixed volume ratio (4:20 *v*/*v*), by adding HF. Interestingly, it was observed that the reflection minima region changed from short-wavelength regions to long-wavelength regions as the HF concentration increased from 8% to 14%. It is known that low-reflection regions shift toward long-wavelength regions with the increasing period of nanostructures [5-8]. The reflectance measurement result reveals the fact that HF concentration affected the period of the Si nanostructures. In other words, high HF concentration increased the period of the resulting Si nanostructures.

To investigate the effects of HF concentration on the period and height of Si nanostructures produced by MaCE, a number of structures within a unit area and average height were roughly estimated from SEM images. With increasing HF concentration, the counted number of structures decreased, which means that the period of the fabricated Si nanostructures increased. This is primarily due to the enhancement of lateral etching of Si MaCE because the lateral etching of Si can be enhanced by increasing HF concentration, when the oxidant is sufficient for providing extra positive holes (h^+^) from the etching front (i.e., metal/silicon interface) to the side of the already formed Si nanostructures [11,15]. Hence, the nanostructures can disappear without distinguishable structure formation, leading to the period increases, if the lateral etching is larger than the radius of the nanostructures [11]. The average height of the Si nanostructures increased from 308 ± 22 to 1,085 ± 147 nm as the HF concentration increased. This is due to the fact that the overall etching rate was influenced by the removal of oxidized Si by HF when the oxidant was sufficient for generating oxidized Si [15]. For this reason, the measured hemispherical reflectance decreases as the HF concentration increases. It is worth noting that the calculated SWR increased from 5.20% to 7.62% as the HF concentration increased from 8% to 14% even though the height of the Si nanostructures much increased. This is mainly because the main energy density region of the solar energy spectrum is located in the short-wavelength region (around 500 nm). This indicates that the HF concentration is crucial for obtaining Si nanostructures with desirable distribution for practical solar cell applications.

**Figure 4:**
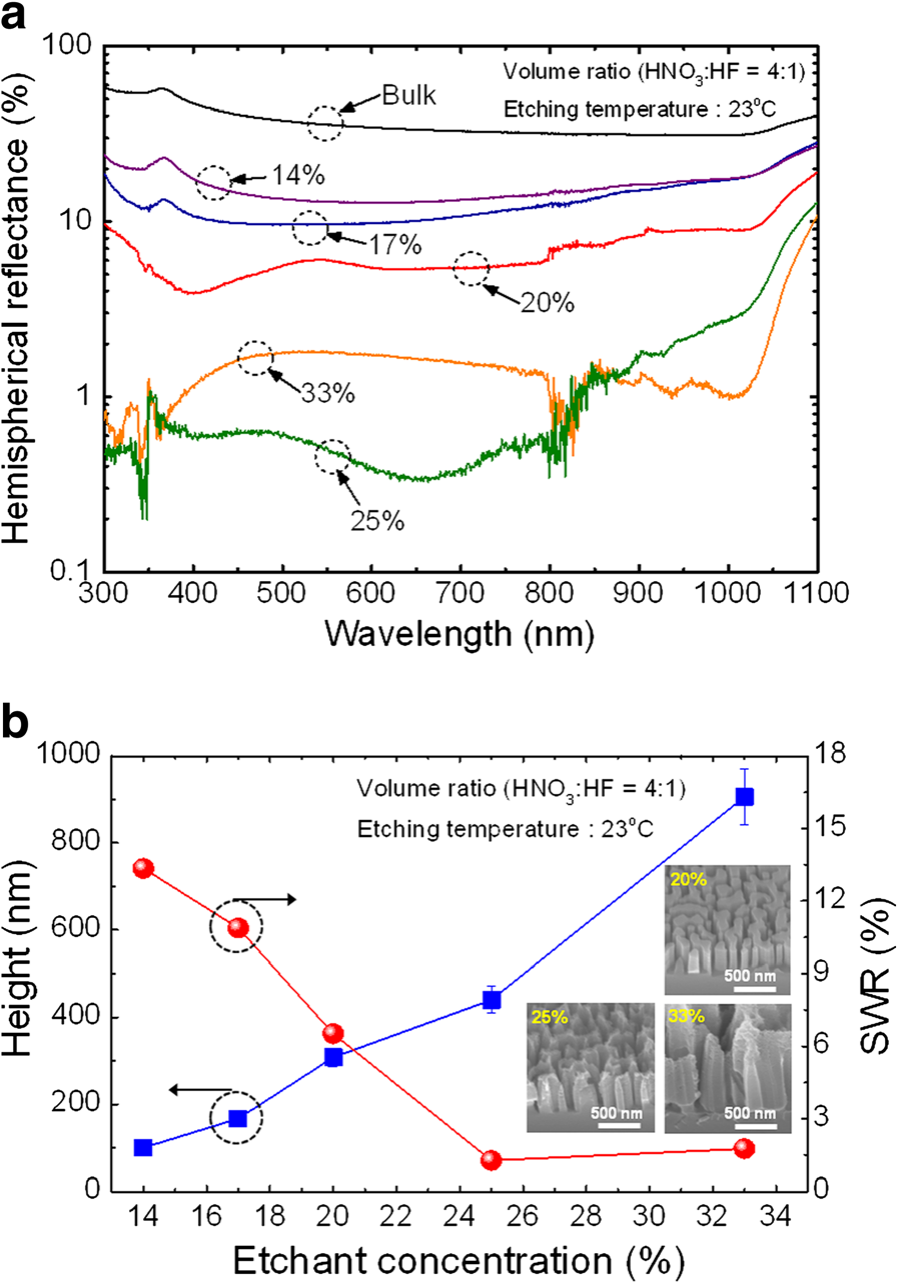
**Measured hemispherical reflectance spectra of Si nanostructures and estimated average height and calculated SWRs.** (**a**) Measured hemispherical reflectance spectra of the corresponding Si nanostructures fabricated using different etchant concentrations from 33% to 14% in an aqueous solution. (**b**) Estimated average height and calculated SWRs as a function of the concentration of etchant. The insets show 45° tilted-view SEM images for etchant concentrations of 20%, 25%, and 33%.

Figure 4a,b shows the measured hemispherical reflectance spectra and the average height and calculated SWR of the resulting Si nanostructures depending on the etchant concentration (i.e., different quantities of DI water). The etchant concentration was adjusted from 14% to 33% in an aqueous solution by adjusting the quantity of DI water while fixing the volume ratio of HNO_3_ and HF (4:1 *v*/*v*). As the etchant concentration increased from 14% to 33%, the average nanostructure height increased from 100 ± 13 to 937 ± 127 nm. This is because of enhanced injection of positive holes (h^+^) into Si and removal of oxidized Si with the increasing etchant concentration [11,15]. As shown in the insets of Figure 4b, the Si nanostructures fabricated using high etchant concentration (e.g., 33%) exhibit severely rough morphology due to excessively high etchant concentration. Although the Si nanostructures fabricated with etchant concentration higher than 25% exhibited a low SWR value of <3% in the wavelength range of 300 to 1,100 nm, the rough morphology is not favorable for practical solar cell applications [10]. From this point of view, the etchant concentration is also very important for obtaining a desirable surface morphology and height of Si nanostructures. Therefore, the etchant concentration of 20% is considered as a potential candidate to produce Si nanostructures for solar cell applications because this condition can produce Si nanostructures with smooth etching profile and a low SWR value of 6.39% in the wavelength range of 300 to 1,100 nm.

**Figure 5:**
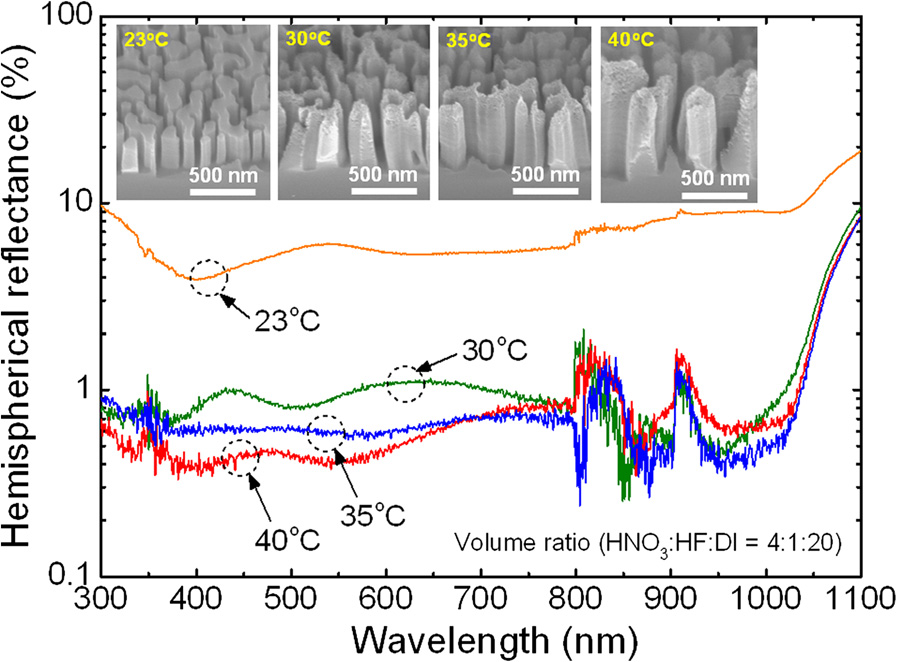
**Hemispherical reflectance spectrum measurement of Si nanostructures.** Measured hemispherical reflectance spectra of the corresponding Si nanostructures fabricated using different etching temperatures from 23°C to 40°C in an aqueous solution composed of HNO_3_, HF, and DI water (4:1:20 *v*/*v*/*v*). The insets show 45° tilted-view SEM images for the corresponding Si nanostructures.

The etching temperature of MaCE is also an important parameter for obtaining Si nanostructures with proper morphology and etching rate. Figure 5 shows the antireflection properties of Si nanostructures as a function of etching temperature. The insets exhibit 45° tilted-view SEM images of the corresponding Si nanostructures. In this experiment, an aqueous solution containing HNO_3_, HF, and DI water (4:1:20 *v*/*v*/*v*) was used. The average height of the Si nanostructures increased from 308 ± 22 to 668 ± 94 nm by elevating the etching temperature from 23°C to 40°C. This result originates from the promotion of carrier diffusion, oxidation, and dissolution during the Si MaCE process at elevated temperature [11,15]. It is observed that the morphology of Si nanostructures is more rough as the etching temperature elevates over 30°C. Although the hemispherical reflectance spectra of the Si nanostructures fabricated using an etching temperature higher than 30°C exhibited lower reflectance and SWR (<1.10%) than the one with an etching temperature of 23°C, they are undesirable for solar cell applications because of their rough morphology. Therefore, careful attention to the etching temperature for Si MaCE is required to produce proper Si nanostructures for device applications.

**Figure 6:**
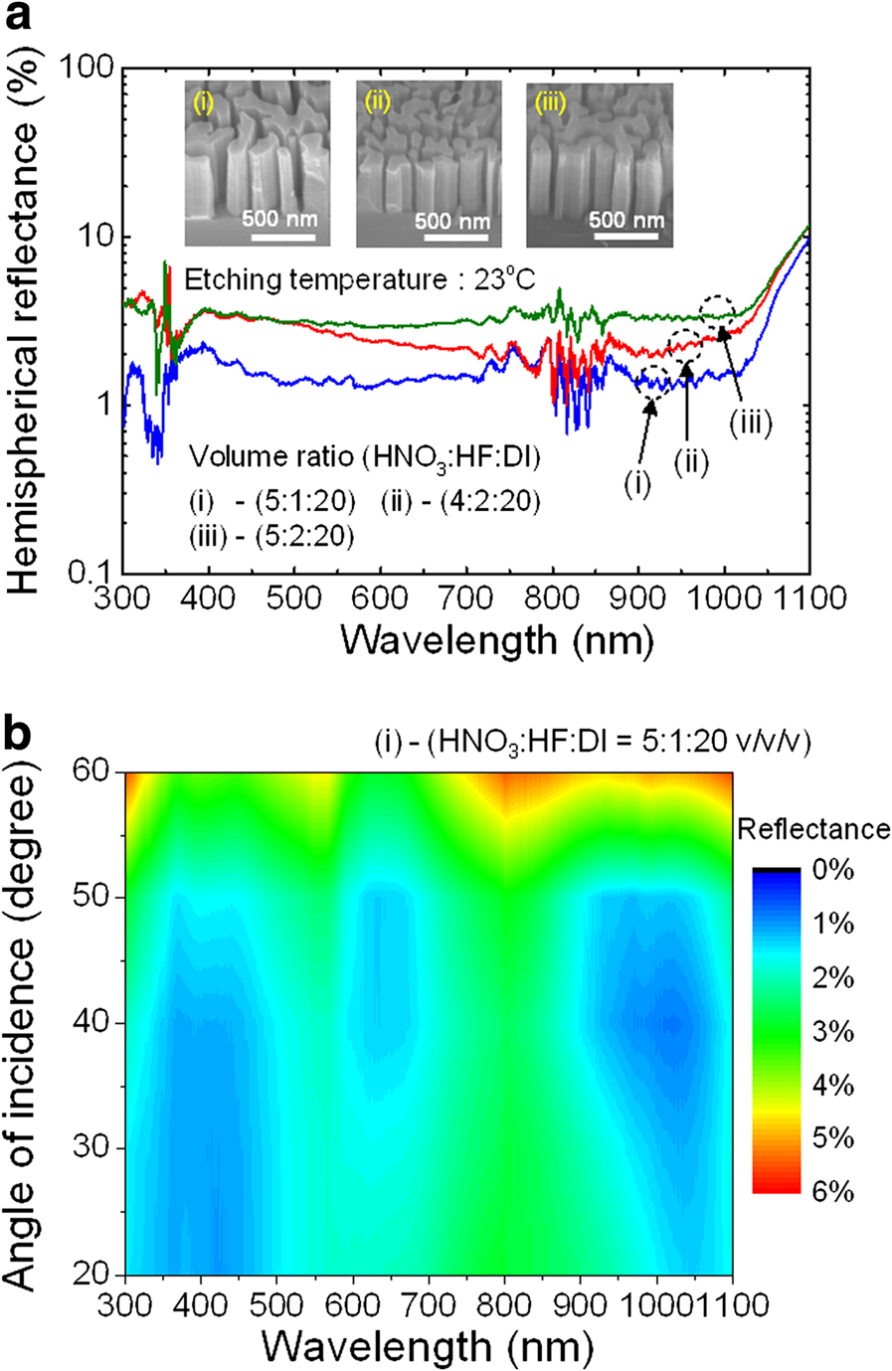
**Hemispherical reflectance spectra and incidence-angle-dependent reflectance as function of AOI and wavelength of Si nanostructures.** (**a**) Measured hemispherical reflectance spectra of the Si nanostructures fabricated using Si MaCE conditions with the HNO_3_, HF, and DI water volume ratios of (i) 5:1:20 *v*/*v*/*v*, (ii) 4:2:20 *v*/*v*/*v*, and (iii) 5:2:20 *v*/*v*/*v* at an etching temperature of 23°C. The insets show 45° tilted-view SEM images for the corresponding Si nanostructures. (**b**) Incidence-angle-dependent reflectance as a function of AOI and wavelength for the Si nanostructures fabricated using condition (i).

So far, we have carefully adjusted the concentration of HNO_3_, HF, and DI water as well as the etching temperature, one by one, to achieve the optimum Si MaCE condition resulting in desirable Si nanostructures for practical solar cell applications. In order to obtain further optimized Si MaCE conditions, we performed an additional experiment using selected MaCE conditions, which are expected to produce Si nanostructures with significantly low SWR and proper morphology as well as etching rate. A Si MaCE process using various aqueous solutions with the HNO_3_, HF, and DI water volume ratios of (i) 5:1:20 *v*/*v*/*v*, (ii) 4:2:20 *v*/*v*/*v*, and (iii) 5:2:20 *v*/*v*/*v* was carried out at an etching temperature of 23°C. As can be seen from the insets of Figure 6a, there is no big difference in the average height among the resulting Si nanostructures (497 ± 24 nm for (i), 472 ± 32 nm for (ii), and 523 ± 27 nm for (iii)), and the surface morphologies are clean without any notable roughness. However, the measured hemispherical reflectance spectra of the corresponding Si nanostructures in the wavelength range of 300 to 1,100 nm were somewhat different. Among the three different Si MaCE conditions, the resulting Si nanostructures using the (i) condition demonstrated the best antireflection characteristic with an SWR value of 1.96% in the wavelength range of 300 to 1,100 nm. This SWR is much lower than that of the pyramid-textured and SiN_*x*_-coated Si surface [22]. This demonstrates the excellence of Si nanostructures produced by MaCE as an antireflection surface for solar cell applications. For stable light absorption of solar cells during daytime, the angle-dependent antireflection property is crucial. Figure 6b shows the contour plot of the incident-angle-dependent reflectance for the Si nanostructures fabricated using the optimum Si MaCE condition of (i), as a function of the angle of incidence (AOI) and wavelength. To obtain angle-dependent reflectance, a Cary variable angle specular reflectance accessory in specular mode was utilized. Although the measured reflectance increases as the AOI increases, the reflectance remained below 6% in the entire wavelength range of 300 to 1,100 nm. The angle-dependent SWR remained below 4% up to an AOI of 60°, while the bulk Si showed an angle-dependent SWR of 37.11%. Thus, the produced Si nanostructures hold great potential for solar cells.

**Figure 7:**
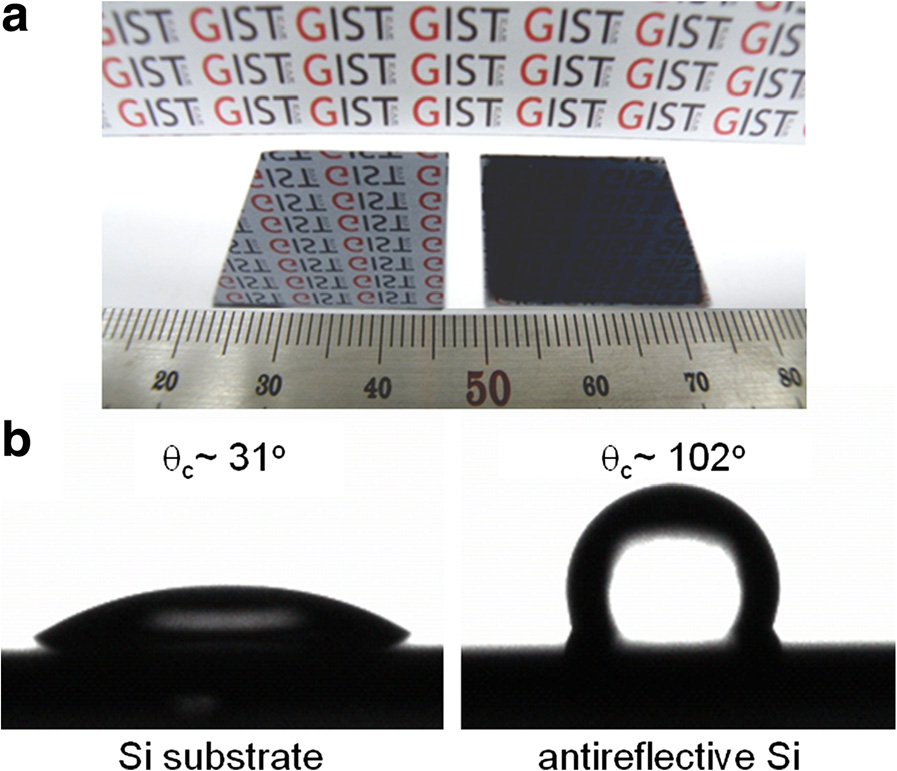
**Photograph and water droplets with a contact angle.** (**a**) Photograph and (**b**) water droplets with a contact angle for bulk Si substrate (left) and antireflective Si (right) fabricated by an optimum Si MaCE condition, respectively.

Figure 7a shows the photographs of bulk Si (left) and antireflective black Si (right) fabricated using the optimum MaCE condition. The bulk Si reflects the background image due to its high surface reflection. In contrast, the antireflective black Si does not reflect anything due to its excellent antireflection characteristics. Figure 7b shows the photographs of water droplets with a contact angle (*θ*_c_) on the surface of bulk Si (left) and antireflective black Si (right). The contact angles of a water droplet were measured using a contact angle measurement system (Phoenix-300 Touch, SEO Co., Ltd., Suwon, South Korea). The bulk Si exhibited a hydrophilic surface with the contact angle of approximately 31°, whereas the antireflective black Si exhibited a hydrophobic surface with the contact angle of approximately 102°. These surface wetting results may be explained by the Cassie-Baxter model [23]. It is known that the hydrophobic surface provides a self-cleaning function, leading to the removal of accumulated dust particles from the surface of solar cells in real environments [19]. Therefore, the Si solar cells with antireflective nanostructures fabricated by the Si MaCE process can achieve much improved efficiency and maintain their early efficiency longer than one with a flat surface.

## Conclusions

We investigated the influence of Si MaCE conditions, including the concentration of HNO_3_, HF, and DI water as well as etching temperature, on the morphologies and optical properties of the fabricated Si nanostructures to achieve the optimum Si MaCE condition, resulting in desirable antireflective Si nanostructures with self-cleaning function, for practical solar cell applications. The optical properties of the fabricated Si nanostructures were strongly correlated with Si MaCE conditions. The Si nanostructures fabricated by an optimum MaCE condition demonstrated the extremely low SWR of 1.96% and an angle-dependent SWR of <4% up to an AOI of 60°, compared to that of bulk Si (SWR, 35.91%; angle-dependent SWR, 37.11%) in the wavelength range of 300 to 1,100 nm, as well as a hydrophobic characteristic with a water contact angle of approximately 102°. These results provide improved understanding of Si MaCE and guidelines to achieve desirable antireflective Si nanostructures with self-cleaning capability for high-efficiency c-Si solar cells.

## Competing interests

The authors declare that they do not have competing interests.

## Authors’ contributions

CIY proposed the original idea, carried out most of the experimental works associated with fabrication and characterization of samples, analyzed the results, and prepared the manuscript. JBK assisted in the experiments and measurements. YMS helped in the characterization of samples and preparing the manuscript. YTL developed the conceptual framework, supervised the whole work, and finalized the manuscript. All authors read and approved the final manuscript.
